# Social Participation and Depressive Symptoms Among Chinese Retirees

**DOI:** 10.1093/geroni/igaa057.1428

**Published:** 2020-12-16

**Authors:** Ziyao Xu, Na Sun

**Affiliations:** Miami University, Oxford, Ohio, United States

## Abstract

With the rapid growth of the older population in China, the number of retirees is expanding. The retirement transition can lead to the loss of roles/challenge self-identity, increasing the risk of depression. Social participation may have a protective role in the context of retirement. The objective of this study was to examine the relationship between social participation and depressive symptoms among Chinese retirees. Using cross-sectional data from the 2015 China Health and Retirement Longitudinal Survey (CHARLS), we investigated 1949 retirees (Mean age = 66.11, SD=0.37). Social engagement was measured by summing participation in 10 social activities (e.g., interacting with friends, volunteering). Depressive symptoms were measured using the CESD-10; a score ≥ 10 was used to define depression. Participants, on average, engaged in 1.42 (SD=0.06) social activities and 16.8% of them had depression. Results of logistic regression indicate a significant relationship between social participation and depressive symptoms (p=0.014). For every additional social activity, the odds of depression decreased by 17%, holding age, gender, marital status, education, total income, and numbers of chronic diseases constant. Social activity is associated with depression among Chinese retirees. Future studies should explore the effects of specific types of activities on the mental health of Chinese retirees.

